# Sanitary Instruction for the Masses
**Conférences sur l'Empirisme*. Par A. Trousseau, Professeur à la Faculté de Médecine de Paris. 1862.*Le Travail, son influence sur la Santé*. Par A. Bouchardat, Prof. d'Hygiène à la Faculté de Médecine de Paris. 1863.


**Published:** 1863-04

**Authors:** 


### Art. XI.—SANITARY INSTRUCTION FOR THE MASSES.*

The two pamphlets referred to below fairly indicate that sanitary instruction is among the things that are managed "better in France" than in our own country. These pamphlets contain lectures delivered by their distinguished authors to the labouring classes of Paris, under the auspices of the Polytechnic Association for the gratuitous instruction of artisans. We know nothing of the Association beyond the present examples of its fruits; but from these we cannot withhold our testimony to their singular merit and great utility.

The lectures of M. Trousseau are evidently published as they were delivered, without a word of preface or comment; while those of M. Bouchardat are followed by a copious appendix of notes and quotations, rendered needful, or at least useful, by the mass of details upon which his deductions are necessarily based. Dealing rather with facts than arguments, it has been his object to enable his readers to investigate these facts for themselves, and to follow them to original sources of information. M. Trousseau, on the contrary, has chiefly to argue and to illustrate; and his lectures are complete in themselves.

By both writers we are forced to the conclusion, either that they must have been levelled much above the heads of their audiences, or else that the Paris *ouvrier* is better educated, and more accustomed to reflection, than his brethren on this side of the Channel. According to our experience, the English artisan, even when he has abandoned the pot-house in order to seek recreation in the acquisition of knowledge, is more prone to develop the observing, than the thinking faculty, more likely to become a naturalist or a collector, than to turn his attention to literary or purely intellectual pursuits. A lecturer addressing the members of a mechanics' institute would seek his illustrations from phenomena visible in the present, in preference to matters requiring for their appreciation considerable knowledge of the past. The gradual character of all development would be shown, to such an audience, by the progress of animal life, or of comparatively recent and well-remembered mechanical discoveries, rather than by the growth of language, or the history of thought. Yet hear M. Trousseau:—

"Observe, gentlemen, if you will look back a little, our beautiful language extricate itself from the swaddling clothes of the

---

* *Conférences sur l'Empirisme.* Par A. Trousseau, Professeur à la Faculté de Médecine de Paris. 1862.

*Le Travail, son influence sur la Santé.* Par A. Bouchardat, Prof. d'Hygiène à la Faculté de Médecine de Paris. 1863.

Mediæval period; observe the struggling of our first writers between the latter, that barbarizes, destroys, crushes them—the German, that pours in from the other side of the Rhine—the Celtic, from the side of the ocean. Observe the stammering, the uncertainties of language, and you will see that successively, from Froissart to King Louis XI., from King Louis XI. to Rabelais, from Rabelais to Marot, from Marot to Ronsard, from Ronsard to Montaigne, from Montaigne to Malherbe, from Malherbe to Corneille, they reach that grand period of the language which the illustrious and immortal Moliere has fixed; that brilliant rest which makes the French of Moliere the most grand and the most pure of all languages spoken by all the world."

More striking, perhaps, than this passage, or even than the frequent classical allusions of both the writers, is the frequent occurrence of illustrations taken from English literature and history, both ancient and contemporary. The sayings of Benjamin Franklin, the statements of Macaulay, about touching for the king's evil, the training of English pugilists, the dietary of English "navvies;" all are briefly glanced at or referred to, and as if entirely within the cognizance of the auditory. It is customary to consider the French ignorance of England, and of things English, perfectly invincible; and this tacit assumption of considerable knowledge appears very worthy of remark. If it have any foundation at all in fact, it may be contrasted advantageously with the mental state of a worthy Dorsetshire farmer, to whom in 1851, we described some portion of the articles shown by Paris manufacturers in the then great Exhibition. "Paris," exclaimed deliberately the *fortunatus agricola*, "Paris be where the Vrenchmen live, ben't it?" We replied that "Some of them did unquestionably live there." "Ah! so I've heerd tell," was the response. "*Be they black?*"

A recent number of the *London Review* contained a description of a piece called *La Prise de Pekin*, played at one of the minor theatres of Paris a year ago. The author had included the Editor of the *Times* among his *dramatis personæ*, and had represented him as living with a Zouave regiment, and as accompanying them into action in his capacity of an observer. "The affection they bore him induced them on more than one trying occasion to press him to withdraw from the front; but his national pride and his strong sense of duty invariably prompted him to resist their friendly importunities, and wherever the enemy's fire waxed hottest, there did the Editor of the *Times* pitch his camp-stool, observing, with an atrocious Fleet-street accent, 'Nong, je ne voo pas me rétirer; il fo que je fasse mon correspondance pour mon journal.' When a fragment of a shell carried off the crown of his straw hat, he philosophically observed, 'Hooroosement il ne fait pas froid;' and when a rifle-bullet twitched his pen from betwixt his fingers, he quietly said, 'C'est egal, j'ai un autre dans mon poche,' and producing it, concluded his letter in time for the home mail." To this description the writer adds:—

"The audience, composed entirely of the middle and lower classes, were charmed with the English editor's coolness and pluck. When, after his capture by the Chinese, he persisted, in the intervals of torture, in explaining at considerable length to his tormentors that 'L'Angleterre était la première pays du monde,' the *bravos* were deafening; and when, towards the close of the fifth act, he paused, on his way to execution, to sing with a manly voice a couple of stanzas of 'God save the Queen,' the admiration of the house broke out in thunders of applause, such as we never before heard in any theatre.

"It was very pleasant for English eyes to see and for English ears to hear all this; the fun the Parisians made of us was so fair and so good-natured, and the praise they awarded to our slow but steadfast courage was so hearty and unreserved. Recalling to mind the stupid, brutal, boozy 'Goddams,' the stage Englishmen of the Boulevards in former days, and comparing them with the brave, intelligent, well-favoured gentlemen who, in 1861, represented our nation on the French stage in their stead, we felt that France and England were at length beginning to appreciate and understand one another; that the *entente cordiale* was becoming something more than an ironical phrase, and that the best blood of the two nations had not been commingled in vain on the stony ridges of the Crimea, and in the muddy waters of the Peiho."

In their very different fashion, and from their very different point of view, MM. Trousseau and Bouchardat convey a similar impression. Their lectures are characterized by a hearty and discriminating approval of some of the best parts of our national character and institutions; and it is evident that they expected their approval, which is conveyed chiefly in brief allusions, to be thoroughly shared by their hearers. The facilities afforded by liberty for scientific investigation, and its influence as a stimulant to labour, are positions more than once illustrated by references to English history, character, or customs.

M. Trousseau opens his subject by an exposition of the difficulties that beset him in his endeavour to speak, of matters essentially medical, to a non-medical audience, before whom he is deprived of his vocabulary of familiar technicalities. He then proceeds to lay down the general proposition that all science has had its origin in empiricism, using the word strictly in the sense of experience, and tracing the cause of this experience usually to some accidental combination of events, such as the use of cinchona by some suffering from intermittent fever. On isolated facts thus obtained, M. Trousseau considers the whole fabric of scientific induction to be based; and he claims for the physician the right to experiment upon his patients, under the restrictions imposed by his knowledge of the experience of past generations—that is to say, when analogy affords a prospect of success without apparent risk, or when death appears imminent, and not to be prevented by ordinary or recognised methods.

In illustration of the last position, M. Trousseau relates how he was summoned, in conjunction with MM. Guersaut, Blache, and two other physicians, to see a child four years of age, apparently in the last agonies of croup. Tracheotomy was suggested, but the case was considered so hopeless, that no one cared to perform it. The mother was therefore told that all treatment would be abandoned, and that an operation—the only hope possible—would not be attempted, as not affording one chance in a thousand of success. At these words she darted to the door, closed it, placed herself against it, and exclaimed, "You will not leave here until the operation is performed." It was done, and the little patient is now alive.

After dwelling with considerable force and clearness upon the impossibility of experimenting usefully, unless guided by such complete knowledge of the natural history of the malady, and of the various methods by which it has been opposed, as may prevent, on the one hand, excursions over ground already trodden, and, on the other, mistaken conclusions about cause and effect, M. Trousseau proceeds to show that herein resides the difference between philosophical empiricism and the empiricism of charlatanry. The physician experiments rationally, safely, with some determinate end in view, and enlarges the boundaries of knowledge. The charlatan experiments at hazard, dangerously, and whether he succeed or fail, does not understand the conditions before him, and with his next patient experiments again *de novo*.

How little the difference thus based upon ignorance or knowledge is understood, even by men of intelligence, is illustrated by the following anecdote of Béranger, whose friend and physician M. Trousseau was for many years:—

"In 1848, Béranger had a slight ophthalmia, for which M. Brettonneau prescribed a collyrium. This cured the ophthalmia; but as Béranger read and worked much, and as his skin was unhealthy, the disorder returned. He applied then to a Polish priest, who treated diseases of the eyes by a secret remedy. At that time I was president of the examiners of *officiers de santé*. The priest had been in difficulties with the police about certain eyes that he had damaged, and wished to secure himself against the law. For this purpose he sought Béranger, and asked the aid of his influence, that he might be admitted an *officier de santé*, and enabled to treat diseased eyes, and to make people blind, in comfort. Béranger came to me, and said: 'Do me the great favour to try and admit this poor devil, who only treats diseases of the eyes; and although the examination of an *officier da santé* includes all branches of the healing art, be indulgent in this case. He is a refugee, and he has cured me—that is the best of reasons.' I replied: 'Send your man to me.' The Polish priest presented himself. 'You are recommended,' I said to him, 'by one whom I greatly desire to oblige—by my dearest friend—in a word, by Béranger. Two of my colleagues, to whom I have spoken, and myself, will do for you whatever is possible; and although our examinations are public, we will perhaps close our ears a little.' I added: "See—I will take the examination in anatomy; and that it may be easy for you to know as much anatomy as myself, I will question you upon the eye.'

"The man seemed disconcerted. I continued: 'You know what the eye is?'—'Very well.'—'You know the eyelid?'—'Yes.'—'Have you an idea what the cornea is?'—He hesitated. 'The pupil?'—'Ah, Monsieur, I know the pupil well.'—'Do you know what is meant by the crystalline lens, the vitreous humour, or the retina?'—'No, Monsieur, such knowledge would be useless to me—I only treat diseased eyes.' I answered that it would be of some use, and a knowledge of the crystalline lens of more than he suspected, if ever he wished to operate for cataract. 'I do not operate.'—'But the fancy might take you to extract one.' He could not escape from that. The miserable wretch wished to exercise the art of the oculist, without having the smallest notion of the anatomy of the eye.

"I went to seek Béranger, and told him the circumstances. He exclaimed, 'But the poor man!' I replied, 'My dear Béranger, I have been your physician for eight years, and to-day I come to ask for my fees.' 'And what fees?' 'You shall write and dedicate to me a song, of which I will give you the *refrain*.' 'Yes, indeed; and the *refrain*.' 'Ah! que les gens d'esprit sont bêtes.' It was an understood affair between us henceforth; and he spoke no more to me of his Polish priest. But it is sad that such things can be told of a man like Béranger, and that he should be unable to see that his *protégé* might do much mischief, and could do no good, even in the most simple maladies of the eye."

M. Trousseau then proceeds to describe, *seriatim*, the leading quackeries of modern times, exposing the devices of their various professors with unsparing hand, and covering them with ridicule that becomes more poignant from its very playfulness. Even the august patronage of the Tuileries does not shield the so-called "mediums," the spirit-rappers, from his pitiless lash; and the fashionable homœopath is rendered famous by the side of the humble bone-setter. We regret that space forbids us to quote M. Trousseau's anecdotes, or to give specimens of his quaint and telling humour. The facts and arguments upon which he relies are only those that are familiar to practitioners in this country; but they have seldom before been clothed in a form so likely to recommend them to the public, or so well calculated to lead up to the right deductions from their teaching. We must, at any rate, find room for the concluding passages of the second lecture.

"If a sudden accident should occur to the powerful engine that keeps in movement the machinery of some vast workshop; if the fuel, the food of the engine—if the steam boiler, that may represent the heart or the blood—if the framework, or the levers, that are in some sense analogous to limbs—be any of them either wanting or broken, everything in that workshop reverts to the silence of the grave! Farewell to the luxury of the master, farewell to the comforts of the workman! Let me then give you advice. Go forth quickly, and seek in the wine-shop for some drunkard, that you may appeal to him to repair the damaged engine.

"But no, you would not go there, gentlemen, no more than, if on board a ship struggling with violent winds, and approaching perilous breakers, you would seek some obscure passenger from the steerage, and intrust him with the command. You would remain silent, and none of you would assume the trumpet of the captain, or endeavour to be heard over the roar of the tempest. You would admit the presence of one who understood his work, and you would confide in him for the safety of yourselves and of your properties.

"But, gentlemen, the body of man, the work of God, is it, do you think, a machine less complex, less perfect as a machine, than any work of human genius? Among you, who nobly earn your means of livelihood by labour, if sickness come to the domestic hearth, then farewell to your innocent pleasures—rewards of the past, anticipations for the future. Daily you see your goods swallowed up in the gaping abyss of usury, daily your wife will deprive herself, even of her clothing, to seek for you the remedy that you need, and to pay the quack who sells it its weight in gold! Ah! gentlemen, do not wait until the greedy empiric has taken the last jewel of the artisan—the ring of betrothal—in exchange for a deceptive and dangerous medicine; but seek the physician who exercises his calling honestly, who has studied it, and who, if he be not so fortunate as to cure you, will at least be able to give you consolation."

The lectures of M. Bouchardat are necessarily more didactic and less rhetorical; in short, more "dry" than those of his eminent colleague. But they contain, clearly and concisely, the popular physiology of industry and idleness; and, in his denunciation of the latter, more especially in his description of the results of monastic idleness in Spain, the author becomes positively eloquent. Speaking for the instruction of the manual labourer, he judiciously dwells upon the value of intellectual work, upon the corporeal effort it entails, upon the repose and the repair that it requires. He sketches the effects of labour in childhood, manhood, and old age, shows the hurtful partial development that may be produced by the continued repetition of the same effort, explains the evils of overwork, and describes how the bad effects of excessive or of partial labour may be, as far as possible, diminished or prevented. Finally, he rises from the particular to the general, from the individual to the nation, and points out the influence of industry or idleness upon the rise or fall of states or empires. The appendix contains figures and quotations infinitely various, authorities chemical, historical, statistical, and legal, for the statements put forth in the lectures themselves; and adapts them to the wants of readers who can inquire for themselves into the cogency of M. Bouchardat's reasoning, and the force of his conclusions.

In looking round us, we can see or recall nothing of equal value in this country. There is scarcely a town in England in which lectures are not frequently delivered, either by some stammering amateur, to a scanty and yawning audience, or by some professional "orator" to an assemblage of ribbons and crinoline, sparsely escorted by the most confirmed male bores of the locality. The amateurs apply themselves to the monuments of ancient Eygpt, or to a cruel murder and dissection of some book of travels or adventure; unless, being clerical, they strive to blend the mildly facetious with the ethically correct, scattering pearls from Joe Miller upon a fabric suggested by the essays of A. K. H. B.; or, being medical, condense the unforgotten portion of the physiology of their student days into something entitled, *The Life of the Four Seasons,* or *The Wonders of the Human Eye.* The professional orator flies at higher game, selects some individual or some subject that has, in comparatively recent times, afforded materials for sharp controversy; and attracts both sides of the question, in order to hear which of them will receive the sanction of the sonorous sentences that roll glibly off his tongue. When very well done, when the carefully written speech has been as carefully committed to memory, and its points are delivered so as to tell, the result is sometimes far from unpleasing, and, if not in the highest style of art, may yet bespeak considerable cleverness. It would not occur to any one who was not fortified by a constitutional admiration of the sound of his own voice to earn a living in this manner, and therefore benevolent persons in the audience may overcome the heartache they would otherwise feel for the hard lot of a man condemned to repeat continually that fine passage about the pendulum, or that splendid description of the sunset, as if it were his casual clothing in words of an idea just presented to his mind. But the professed lecturer usually seeks to fascinate by ornament and antithesis rather than to instruct by the careful sifting of evidence; and the amateur too often has himself "got up" some subject on which he is not qualified to speak by the fulness of his mind, and, in his account of which, the dry husks of grinding painfully predominate. In either case, that which is conveyed to the audience passes through their heads like water through a pipe. We have ourselves listened with pleasure to the most finished lecturer of the day descanting upon the life and times of Curran, and we only remember that at some period of his existence he was called Orator Mum! We have ourselves, at various times, striven to popularize fragments of science for country town audiences, and we never found a hearer, not even among the "intelligent committee," who could tell, a week after the performance, how the solar spectrum was modified by the presence of colloids, or what influence vegetable parchment exerted upon the dialysis of the sodium line. But such lectures as those of MM. Trousseau and Bouchardat are rare indeed. Forcible, simple, authoritative, spoken worthily of masters of our art, and dealing with facts and questions of continually recurring interest in daily life, they are likely to bring home the truths of sanitary science, as far as they embrace them, to the convictions and the understandings of their hearers, while the lecturers, removed by their position from mere spouting for gain, or the mere gratification of vanity, are at once felt to be engaged in a task undertaken for the public good, and only to be rewarded by the public gratitude.

We should hail with pleasure, in our own country, some imitation of so excellent an example. Sanitary evils have their foci in the homes of the artisan class, and while the members of this class are ignorant of, or indifferent towards, them, they can never be completely removed. Until the labouring man demands a wholesome habitation for himself and his children, the reeking dens of our great cities, the two-roomed cottages of our agricultural districts, will still be crowded by unhealthy and demoralized inmates; and until he comprehends some of the truths that M. Trousseau has so eloquently expressed, he will still be the victim of ignorant druggists and extortionate quacks, who in many parts of England live in affluence upon his credulity. The support of quackery by the upper classes from their superfluities is in many respects a less evil than that from out of the scanty pittance of the workman; and in the upper classes the evil must be left to die out before the increasing skill and knowledge of the profession. Among the poor, however, both with regard to this and to other sanitary evils, there is much room for the active antagonism of the teacher; and where amateur lecturers of sufficient personal knowledge and sufficient wealth of language to deal adequately with such subjects are not forthcoming, we can only most cordially recommend to others the discourses of MM. Trousseau and Bouchardat as affording both materials for argument and models for imitation.

---

